# Structural barriers and facilitators to accessing HIV services for marginalized working populations: insights from farm workers in South Africa

**DOI:** 10.1093/heapol/czae098

**Published:** 2024-10-26

**Authors:** Nosimilo Mlangeni, Martina Lembani, Olatunji Adetokunboh, Peter S Nyasulu

**Affiliations:** Division of Epidemiology and Biostatistics, Department of Global Health, Faculty of Medicine and Health Sciences, Stellenbosch University, Francie van Zijl Drive, Cape Town 7505, South Africa; National Institute for Occupational Health, a Division of National Health Laboratory Service, 25 Hospital Road, Johannesburg 2001, South Africa; School of Public Health, University of Western Cape, Robert Sobukwe Road, Cape Town 7535, South Africa; Division of Epidemiology and Biostatistics, Department of Global Health, Faculty of Medicine and Health Sciences, Stellenbosch University, Francie van Zijl Drive, Cape Town 7505, South Africa; DSI-NRF Centre of Excellence for Epidemiological Modelling and Analysis, Stellenbosch University, Francie van Zijl Drive, Cape Town 7505, South Africa; The University of the People, 595E Colorado Blvd, Pasadena, CA 91101, USA; Division of Epidemiology and Biostatistics, Department of Global Health, Faculty of Medicine and Health Sciences, Stellenbosch University, Francie van Zijl Drive, Cape Town 7505, South Africa; Division of Epidemiology and Biostatistics, School of Public Health, Faculty of Health Sciences, University of the Witwatersrand, 1 Jan Smuts Avenue, Johannesburg 2001, South Africa

**Keywords:** Farm workers, universal health coverage, access to health services, HIV, social determinants of health

## Abstract

Farm workers are vulnerable working populations who face significant inequalities in accessing health services, including those for human immunodeficiency virus (HIV) prevention, treatment and care. This descriptive phenomenological study aimed to explore farm workers’ experiences when accessing HIV services and was conducted in Limpopo province, South Africa. Eighteen in-depth interviews were conducted in four health facilities from two districts, and two focus group discussions were conducted in one of the farms within the province. Purposive sampling and systematic random sampling were used to select study participants. A deductive thematic approach was used to analyse data, informed by the social–ecological model of health. The results reveal that farm workers perceive multiple interdependent factors that inhibit or enable their access to HIV healthcare services. Key barriers to HIV healthcare were transport affordability, health worker attitudes, stigma and discrimination, models of HIV healthcare delivery, geographic location of health facilities and difficult working conditions. Key facilitators to HIV healthcare included the availability of mobile health services, the presence of community health workers and a supportive work environment. The findings suggest disparities in farm workers’ access to HIV services, with work being the main determinant of access. We, therefore, recommend a review of HIV policies and programmes for the agricultural sector and models of HIV healthcare delivery that address the unique needs of farm workers.

Key messages

- Farm workers face unique employment conditions and geographic barriers that limit access to HIV prevention and treatment services.
- Proper linkages to care facilitate retention in antiretroviral therapy for cross-border migrant farm workers.
- Models of care HIV care delivery should be uniquely designed to address the geographic barriers and high-mobility nature of farm workers.

## Introduction

More than 3 billion people struggle with access to essential health services globally (Garchitorena *et al*., 2021), while >80% of the global workforce is without occupational health and safety (OHS) services (Tabibi *et al*., 2018). Based on the universal health coverage (UHC) principles, access to high-quality healthcare should be within reach for all populations, including formal and informal workers and the poor and vulnerable (Richard *et al*., 2016). One of the aims of the UHC is to improve the level and distribution of health and health services (National Academies of Sciences, Engineering, and Medicine, 2016), and advancing equitable access to healthcare is one of the public health responsibilities (Golden and Wendel, 2020). Healthcare access in the context of this study indicates the opportunity to identify healthcare needs, seek healthcare services, reach and use healthcare services and have healthcare needs fulfilled (Levesque *et al*., 2013).

South Africa is one of the countries where health inequalities remain a challenge, as differences in access to healthcare remain within and between different population groups (Arcaya *et al*., 2015; Richard *et al*., 2016). Previous studies have reported higher self-reported poor health among lower socio-economic groups in South Africa (Ataguba *et al*., 2011; Alaba and Chola, 2014). Health inequalities originate from social and economic disparities, and unequal distribution of power and resources (Marmot and Bell, 2010), which is evident among vulnerable workers working in precarious employment. Precarious employment is considered one of the most important determinants of health inequalities and is characterized by employment insecurity, income inadequacy and limited workplace rights and protection (Benach *et al*., 2014; Gunn *et al*., 2022).

Farm workers are one of the vulnerable working populations in precarious employment faced with economic deprivation. They face significant inequalities in accessing essential health services compared to non-agricultural workers (Harwell *et al*., 2022), including OHS, human immunodeficiency virus (HIV) prevention, treatment and care. Long distances to health facilities, conditions of employment, lack of workplace health services and health system barriers contribute to their struggle to access quality care (Munyewende *et al*., 2011; Loganathan *et al*., 2019). Circumscribing access barriers is a first step towards reversing disparities in HIV prevention for farm workers and is an important prerequisite for achieving UHC.

Several research studies have reported on facilitators to HIV healthcare access, particularly for hard-to-reach populations, such as the availability of social support, community health workers (CHWs), mobile clinics and good health provider and patient relationships (Croome *et al*., 2017; Garchitorena *et al*., 2021; Myburgh *et al*., 2021; Harwell *et al*., 2022). However, farm workers are not similar to general rural populations; they have a unique social construct that distinguishes them and their social determinants of health from other populations. Thus there is a dearth of studies reporting on farm workers’ barriers and facilitators to HIV healthcare, particularly in the South African context. Furthermore, farm workers’ access to primary health care (PHC) community services, such as CHWs, and mobile health facilities is not well recorded in the literature.

In 2010, a disproportionately higher prevalence of HIV (35–42%) was reported among farm workers in South Africa (Colvin, 2010; Klaas *et al*., 2018), while that of the general population was 17.9% in the same year (National Department of Health, 2010). They have an increased HIV risk due to several factors, such as their high-mobility status and their likelihood to engage in high-risk sexual behaviour (International Organization for Migration, 2005; Tiruneh *et al*., 2015; Durojaiye *et al*., 2020). The International Labour Organization’s (ILO) Code of Practice on HIV and acquired immunodeficiency syndrome (AIDS) (International Labour Organization, 2001), the South African Code of Good Practice on HIV and AIDS and the World of Work (Department of Labor, 1998) provide guiding principles on the workplace’s role in responding to HIV and AIDS. However, few studies seek to understand the availability of workplace HIV programmes for farm workers, as well as their experiences in accessing HIV services.

Ensuring access to comprehensive HIV prevention programmes as well as treatment and care services is critical to mitigating the impact of the epidemic (Loganathan et al., 2019), especially for highly vulnerable most at-risk populations. Furthermore, understanding a population’s perceptions and felt experiences when needing or utilizing health services plays a crucial role in developing measures to improve equitable access to care. The aim of this study, therefore, was to explore farm workers’ experiences in accessing HIV prevention, treatment and care services.

## Methods

### Study design

A descriptive phenomenological study design was employed, nested in a larger doctoral study that involved mixed methods. This was the most appropriate study design as it is meant to present an individual’s experiences in their own words, and a researcher may move beyond what an individual participant reports by clustering together common ideas from multiple participants (Willis *et al*., 2016).

### Conceptual framework

**Figure 1.**
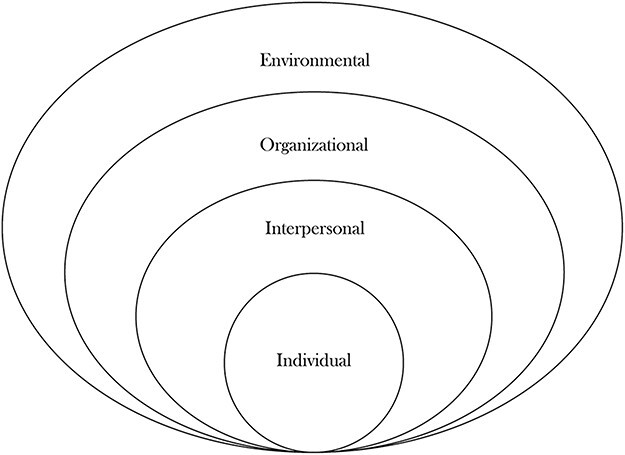
Social–ecological framework on healthcare access for working populations, adapted from McLeroy *et al*. (1988)

Although there are several health access frameworks, the social–ecological model (SEM) was chosen as a conceptual framework for this study (Figure 1). This framework is useful when understanding complex issues such as experiences in accessing healthcare as it helps to visualize individual health within its broad contexts (Golden and Wendel, 2020). Health inequalities cannot be understood independently of social dynamics and institutional and state decisions that play a contributory role towards inequitable rendering of healthcare (Golden and Wendel, 2020). Therefore, using this model provided a better understanding of the interplay of individual and structural factors that impede or facilitate access to HIV healthcare. As such, the SEM has been one of the models used in different settings for studies that examined healthcare access for marginalized populations (Chimphamba Gombachika *et al*., 2012; Garney *et al*., 2021). The SEM model used in this study is adapted from McLeroy *et al*. (1988) and categorizes context into four levels, which are individual, interpersonal, organizational and environmental factors. These four levels of the model demonstrate the interconnectedness of multiple levels of influence in access to healthcare and also provide a platform for multilevel interventions that would effectively improve access to HIV healthcare services for the study population.

### Study setting

**Figure 2.**
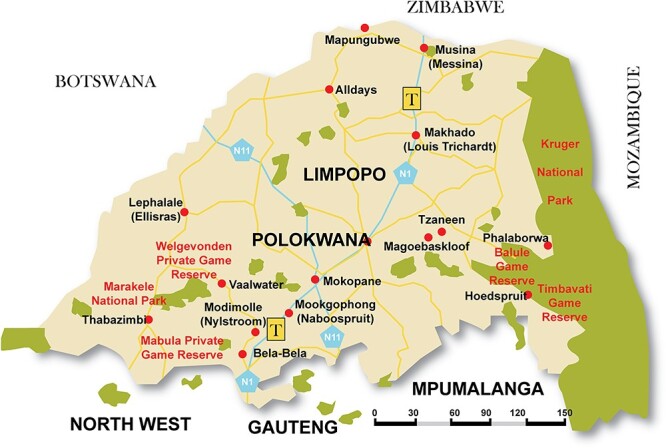
Map of Limpopo province, Wikepedia, 2023

The study was conducted in Limpopo province, South Africa (Figure 2). Limpopo is one of the nine provinces and borders Mozambique, Botswana and Zimbabwe and forms a link between South Africa and countries in sub-Saharan Africa (SSA). The province has rife agricultural activities and is predominantly rural, with >80% of the population living in rural areas (Malatji, 2020). Due to its geographic location, the province is a recipient and home to many local and migrant farm workers.

The study setting was the PHC facilities of the Musina local municipality, which is in the Vhembe district, and the Maruleng local municipality, which is in the Mopani district, as well as the farms in both districts. The selection criteria for the districts were a higher number of cross-border migrant farm workers from neighbouring countries. Four PHC facilities were purposely sampled with guidance from both health districts, who advised on facilities that provide health services to more farm workers. The selection of one participating farm followed purposive sampling, where the criterion was that the farm should be further than a 10-km radius from the nearest health facility.

### Study population and sampling methods

The study population was farm workers in the Mopani and Vhembe districts of Limpopo. Maximum variation purposive sampling was used to select study participants attending the PHC clinics for in-depth interviews (IDIs). Participants had to meet one or more of the following criteria: antiretroviral therapy (ART) patient, cross-border migrant, South African, and man or woman. This criterion allowed the sample to represent views of different categories of farm workers, as it factored in gender, HIV status and migration status. Due to a higher number of participants who met the purposive criteria for focus group discussions (FGDs), systematic random sampling was used to select participants for FGDs from the participating farm. This was achieved by selecting every fifth male and fifth female from workers who volunteered to participate in the study. This approach ensured that all farm workers who were eligible and had volunteered to be part of the study had an equal opportunity to participate in the FGDs, as there were no reasons to choose one participant over the other.

### Data collection instruments

Data were collected using two approaches, which were IDIs and FGDs. The IDIs were conducted during data collection in health facilities. They were considered the best approach that provides privacy because of the sensitive discussion topics being addressed. On the contrary, the FGDs were considered more suitable when collecting data from the farms as the discussion questions were more neutral and not sensitive. Moreover, workers on each farm are familiar with each other, therefore, making it easy to engage and open up in a group setting. The FGDs were added as a second data collection method because first, there was an understanding that some farm workers may not be using the PHC facilities, but they should still be reached with HIV prevention services, such as HIV education, behaviour change communication and condom supply. Thus, this information was to be collected from the farms where those who are not usually found in health facilities could be reached. Secondly, a group setting was going to provide a platform where farm workers could open up about their experiences without feeling like they were being targeted. Lastly, using FGDs provided an opportunity for data triangulation, which strengthened the study results.

An interview guide with semistructured questions informed by the research objectives was used. The interview guide covered the following discussion topics: HIV services that participants were aware of, experiences with healthcare providers, experiences with employers when attending to healthcare, costs and distance to nearest PHC facilities, stigma and discrimination and continuum of care for those on HIV treatment.

Additionally, an FGD guide with semistructured questions, also informed by the research objectives, was used to conduct FGDs. The discussion guide covered the following topics: types of HIV services provided in farms, how HIV prevention services are accessed, enablers and challenges to accessing health services, experiences with healthcare providers and what could be done to improve access.

### Data collection

The study was presented to all clinic attendants in participating health facilities, and farm workers attending health services at the time were invited to participate. Study information leaflets in participants’ languages, which were English, Xitshonga, Tshivenda and Sepedi, were issued to potential participants. Informed consent was collected from those who volunteered to be enrolled in the study. The interviews, led by the Principal Investigator (PI), were conducted in English with the assistance of a multilingual translator. This was done to ensure that participants who were not English speakers understood the questions and answered in their vernacular languages which were translated back to the interviewer in English. The duration of each interview varied depending on the responses, lasting for ∼40 min on average.

FGDs, which followed the IDIs, were conducted in farms following a presentation of the study to the farm manager and workers. Information leaflets were issued to potential participants, and informed consent was collected from those who volunteered to be enrolled. The FGDs were conducted in English, with the assistance of a multilingual translator. Two FGDs were conducted: one group comprising 10 female participants and the other group comprising 10 male participants. Each FGD took 60 min on average.

Interviews were recorded digitally and labelled with a unique code for each participant. All audio recordings were kept on a password-protected computer accessed only by the PI to ensure confidentiality.

### Data analysis

The interviews were transcribed verbatim. Transcriptions were done by different researchers from data collectors, who were multilingual and translated while transcribing. This guaranteed the quality of translations during data collection, as transcribers could ensure that the meaning between translators and participants had not been lost. Initially, the PI and a second researcher coded four diverse transcripts independently, and coding meetings were held to discuss and agree on the codes. A hybrid approach was used to analyse data, starting with a deductive approach in data coding to identify codes using the SEM framework, and then later, an inductive approach to identify any emerging codes and themes not previously identified within the SEM framework, as explained by Fereday and Muir-Cochrane (2006). The SEM framework was flexibly used with the potential for adaptation to demonstrate this study’s findings within the results write-up. The PI conducted a thematic analysis and agreed on the identified themes with the co-investigator.

### Trustworthiness

Lincoln and Guba’s criteria of trustworthiness were applied in the study (Nowell *et al*., 2017). Two researchers independently coded the data, and an inter-coder agreement was reached to ensure the reliability of the findings. Repetitive listening to audio records, and repeated reading of transcripts, allowed for prolonged engagement with the data, thereby ensuring credibility. Narrative extracts were attached as references to support the final generated themes.

## Results

### Participant characteristics

Eighteen participants were interviewed in IDIs (Table 1). Participants consisted of farm workers who have different job responsibilities, the majority being from Zimbabwe (*n* = 8) and South Africa (*n* = 8). Two FGDs were conducted, consisting of 20 participants, with half (*n* = 10) being females while the other half (*n* = 10) being males (Table 2).

**Table 1.** Demographic characteristics of IDI participants

Characteristics	*N* = 18 (100%)
Gender	
Male	8 (44.4)
Female	10 (55.6)
Age (years)	
18–49	12 (67)
>50	6 (33)
Nationality	
South Africa	8 (44.4)
Zimbabwe	8 (44.4)
Swaziland	1 (5.6)
Mozambique	1 (5.6)
Type of work	
Fieldwork	5 (27.8)
Housekeeping and maintenance	6 (33.3)
Security	2 (11.1)
Supervisor	1 (5.6)
Packing and other	4 (22.2)

**Table 2.** Demographic characteristics of FGD participants

Characteristics	*N* = 20 (100%)
Gender	
Male	10 (50)
Female	10 (50)
Age (years)	
18–49	16 (80)
>50	4 (20)
Nationality	
South Africa	9 (45)
Zimbabwe	11 (55)

### Factors affecting access to HIV services

Table 2 presents participants’ experiences when accessing HIV healthcare services according to the SEM framework. The emerging themes were individual, interpersonal, organizational and environmental factors. Sub-themes were identified under each main theme, as presented in Table 3.

### Individual factors

Individual factors are personal characteristics that play a role in an individual’s ability to access health services. Participants expressed reasons for working at the farms, poverty being the main reason, with some participants being cross-border labour migrants.

#### Low socio-economic status

Most participants opted for farm work because of financial difficulties and low education levels. However, farms pay low wages, and earned income does not meet all their family needs. Some participants defaulted on treatment as they could not afford transport fares to the health facilities:

*The situation on my weekly farm work is too difficult because the farm does not pay us enough. So, my family is poor, the money that I earn monthly is not enough for me, but I push myself to continue working because I am not educated enough, I did not finish school*.(IDI 4: migrant male)

Other participants experienced delayed diagnosis and treatment due to transport fare unaffordability:

*For example, if I am one month pregnant and want to get tested, but I cannot come to the clinic because I don’t have money, the following month I still don’t have money, up until three months. That means I will only be screened for HIV 3 months into my pregnancy, which is a bad thing. I must be screened in the first month so that if I have HIV they can initiate me on treatment early in my pregnancy*.(IDI 2: migrant female)

#### Cross-border migrants

Workers who are cross-border migrants mostly reported that poverty pushed them to come to South Africa and work in the farms They send their earnings home every month, but due to low income, they are left with little money, making it difficult to afford healthcare (due to high transport costs):

*What made me leave my country was realizing I needed a job to survive and raise my children. I decided to cross over to South Africa and come to the farms because I heard others say that female farm workers were needed here. But going to clinics is difficult because transport is expensive – it costs me R200 to attend the clinic*.(IDI 5: migrant female)

### Interpersonal factors

Interpersonal factors relate to a person’s interaction with people, which can provide social support or create barriers to accessing health services. Participants shared negative and positive experiences with health providers when using healthcare services. They also shared their experiences of stigma and discrimination.

**Table 3.** Inequalities in accessing HIV healthcare services based on the SEM

Ecological level (McLeroy, et al., 1988)	Theme	Sub-theme
Individual factors	Low socio-economic status	Health service affordability
		Cross-border migrants
Interpersonal factors	Patient–provider relationship	Discrimination based on nationality and language
		Positive health worker attitude
	Stigma and discrimination	Fear of stigma and discrimination
		HIV status disclosure
Organizational factors	accessed HIV services	HIV prevention, treatment, care and support
		Facility operating hours
		Linkages to care
	Models of health delivery	Primary healthcare facility-based services
		Outreach/mobile healthcare model
Environmental factors	Geographic barriers	Financial and physical distance costs
		Treatment default and delayed diagnosis
	Work environment	Working conditions
		Psychosocial support
		Mandatory HIV testing

#### Patient–provider relationship

Some experienced discrimination based on language and nationality, with health workers showing negative attitudes towards those who could not speak a local language. Participants perceived that they needed to be able to speak the health workers’ language to be acceptable; thus, they felt unwelcome to use the health services:

*I was speaking my language and I couldn’t speak hers, then she had that thing of ‘why can’t she understand, why can’t she go to the people who speak her language*. (IDI 18: migrant female)

*So if I tell them no, I can’t speak Shangaan, and I can’t speak Sepedi or whatever they said so what are you doing here? If you don’t know how to speak our language, why don’t you return to your country?*(IDI 17: migrant female)

Other participants reported good experiences when accessing HIV services either at health facilities or through mobile healthcare provided at the farms health workers had a positive and accepting attitude towards them:

*Those people they are good. If we are sick with flu they treat us. Those who need family planning they provide it to them*. (IDI 2: migrant female)

#### Stigma and discrimination

There were experiences of stigma and discrimination for those who live with HIV in some farms. Co-workers would make fun and laugh at those who collect medication from mobile clinics. Due to fear of dismissal based on living with HIV, some participants were afraid to let their employers know about their HIV status. Other participants reported that their colleagues do not like to collect medication from the mobile clinics due to fear of stigma:

*What I like about collecting my medication from the health facility far away from the farm is that at the farm as people come to the mobile clinic they will know that so and so were in the queue to collect HIV medication because there are so many people there and they tell others about people who are taking ARVs*.(IDI 1: migrant female)

*Here in the farm, if you have the HIV disease and people know about it, when you go to the mobile clinic to get your medication people come to see if you are really collecting medication. When you leave they laugh at you and gossip about you, saying ‘she guzzles the pills too’*.(FGD 1: females)

### Organizational factors

Organizational factors include rules, structures and institutions that promote or constrain access to health services. Participants had access to several HIV services, either at the health facilities or through mobile clinics. Models of HIV healthcare delivery were limited to traditional healthcare delivery within the primary healthcare structure.

#### accessed HIV services

Most participants did access HIV prevention services in the farms where they worked. Almost all participants reported that condoms were delivered to tuck shops, while some reported that mobile health services visited their farms once a month. Mobile services provided them with voluntary testing and counselling, initiation to ART for those who tested positive, as well as provision of chronic medication, including ART:

*Mobile clinics deliver the boxes of condoms in the farm, I have seen the condoms there. They have been delivered there, sometimes health workers come and teach us how to prevent HIV*.(IDI 18: migrant female)

*There were people of health who were coming to the farm, so they ask if someone wants to test for HIV…. they asked if there’s anyone who wants to test for HIV, they called for those who wanted to test. Then they said you are HIV positive, do you want to take pills? I said yes, and they started to give me the tablets*.(IDI 4: migrant male)

Participants reported the presence of peer educators and treatment supporters in some farms, who mainly provide support to those who are living with HIV. They regard them as people who help them with health matters. Other participants reported the presence of CHWs, who assist with chronic medication at the farms:

*We get HIV education from the community health workers, they teach us that if you are going to have sexual intercourse you must use protection, like condoms*. (IDI 5: migrant female)

Some participants expressed frustration over long waiting times when collecting medication from the health facilities. Although they may arrive when the clinic opens, they would wait 1–2 h while health workers chatted with their colleagues in the examination rooms. This made them feel that their well-being was not being considered:

*You arrive early, maybe 6 a.m. you are waiting here, only to be attended at 9 a.m. or 10 a.m. They have written that starting hours are maybe 8 a.m., but from 8 a.m. to 9 a.m. nobody is taking care of you. In most clinics they don’t care about the patients, they don’t care about time. The queues outside there, some children are starving, people are suffering in the clinics, to be honest*.(IDI 18: migrant female)

Participants who frequently travelled to their home countries reported that they were able to request more medication when they intended to travel, and they were provided with treatment that lasted until they returned:

*If I know I want to go home for three months, I go to the clinic, and they give me treatment for 3 months to travel with*.(IDI 4: migrant male)

*When I am going to Zimbabwe I make sure to travel with enough pills that will last me for my stay, and I come back when I know it’s time to collect pills*. (IDI 6: migrant male)

Those who came to the country when already on ART reported that they brought transfer letters from their countries of origin, and they continued with similar regimens that they were taking from their home countries, the medication was the same:

*They wrote a transfer letter for me so I could get treatment where I will be working and living. I realized that I can’t be staying here in South Africa and getting medication in Zimbabwe, I may not have transport money to collect my medication from there, so they wrote me a transfer letter*.(IDI 1: migrant female)

#### Models of healthcare delivery

Based on discussions with study participants, there were two models of HIV healthcare delivery for farm workers: the PHC facility-based model and the outreach model through mobile clinics. Most participants in one study sub-district had access to PHC facilities and the mobile clinic. None of the participants in the other study sub-district reported access to a mobile clinic. Those who could not access the mobile clinic had a greater challenge with access to HIV services.

### Environmental factors

Environmental factors comprise a broader context that enables or impairs access to healthcare. The environmental context looked at barriers to HIV services, including distances to health facilities and the work environment, which greatly influence workers’ ability to use HIV healthcare services as and when required.

#### Geographic barriers

Some participants found it difficult to reach health facilities due to the geographic location of farms. They travelled long distances to the nearest health facilities, and some areas did not have transport services. Others had to hitch-hike due to the scarcity of transport or expensive fares:

*The problem that we face is coming to the clinic, hey … hey, coming to the clinic is hard! You see, even after this visit, getting transport to the farm will be a problem because there is no transport. The transport that might be available will require me to buy petrol so that it can take me to the farm*.(IDI 3: non-migrant male)

*From there we pay R100 for transport. If I leave the farm around 05h30 in the morning, I will get here around 08h30, and the total transport cost for a return trip is R200*. (IDI 10: migrant female)

### Work environment

Most participants experienced poor working conditions, worked long hours and only rested when they were on leave. Some participants, especially seasonal workers, said they do not get paid sick leave. Several workers also reported that even though they struggled to get to health facilities, their employers did not attempt to assist:

*Employers from here don’t help anyone with anything. If you get sick at work they will tell you to go home and sit, and your ticket (payment) is at a standstill it does not move. But that is when you are employed temporarily because those on permanent employment when they get sick and cannot work, when they come back their ticket would have moved and mine wouldn’t have moved just because I am a temporary employee. So that is why we think these employers don’t treat people well*. (FGD 2: males)

However, some workers had good working conditions, where they had paid sick and vacation leave:

*If I don’t produce a sick note, there is no pay. But like now I’ve got one, then they will pay me for those hours I’ve spent here. Even when I am going to the hospital, I ask them to give me a day off. They also give me vacation leave*.(IDI 18: migrant female)

One participant had experienced mandatory HIV testing at his farm, where all new employees get tested for HIV as part of pre-employment screening tests. The participant did not know whether the health workers who conducted the tests shared the workers’ HIV results with the employer or not:

*Yes, once you get employed to work in our farm, before you start working you go to the clinic, they test you for HIV and they also test your urine*.(IDI 14: non-migrant male)

Employer support to workers who live with HIV, in the form of transport arrangements or time off to collect medication, was expressed as important by most participants. Those who did not get any form of transport or other psychosocial support from their employers found it more difficult to access healthcare services:

*He gives me money when I tell him that I want to attend the clinic on a particular day, the money helps me to come to the clinic. But he deducts that money from my salary*.(IDI 10: migrant female)

*I work for two people. So I told the other one, and then that one accepted. And sometimes she’s the one who brings me here*.(IDI 17: migrant female)

Disclosure of HIV status to close family members had a positive impact on participants. Those who had disclosed expressed positive feelings about living with HIV and being on life-long therapy. Family support was important in keeping up with treatment collections and adherence. Some participants said they trusted their employers and had disclosed their status to them, and thus received adequate support, which included transport and time off on treatment collection days.

## DISCUSSION

This study aimed to explore the perspectives of farm workers on their experiences when accessing HIV prevention, treatment and care services. Our study found that the most common barriers experienced by farm workers when accessing health resulted from individual and environmental factors. These findings are consistent with previous studies that found that distance to health facilities and affordability are some of the barriers to accessing health services (Peters *et al*., 2008; Tuller *et al*., 2010; Garchitorena *et al*., 2021). The inability to afford HIV services was mostly attributable to distance to health facilities, low income and poverty. Some of the migrant workers got involved in agricultural work due to extreme poverty conditions in their families, and although they were in a better position to provide for their families, they were not left with adequate finances to manage their day-to-day needs.

Workers who reported having mobile services coming to their farms were in a better position to access all HIV services, albeit the frequency of services was low, being once a month in most cases. Those who did not receive mobile health services relied on PHC facility-based services, which enabled geographic and financial barriers. These findings are consistent with previous research findings that indicate mobile health services reduce barriers to accessing HIV treatment, especially in rural areas (Schwitters *et al*., 2015; Gizaw *et al*., 2022). Long distances increased the costs of accessing PHC services for the study population. High transportation costs have been reported to be a potential barrier to sustained ART adherence and retention in care (Tuller *et al*., 2010).

The work environment, especially employment conditions, played a huge role in facilitating access to HIV services in our study. Previous studies have reported that migrant farm workers, especially those on seasonal employment, are likely to lack sick leave benefits (Deblonde *et al*., 2015; Loganathan *et al*., 2019), which limits their access to healthcare. Workers who received support in the form of paid sick leave and transport to health services reported better access to HIV services. However, none of the workers reported the availability of HIV workplace programmes in their farms. According to London, occupational health services can play a role in the control of HIV, if HIV programmes are integrated into general health and safety programmes (London, 1998). Based on the findings of this study, such an approach may improve access to HIV services for farm workers; however, most agricultural settings lack occupational health services (International Labour Organization, 2011).

Although the provision of HIV services in the workplace has been shown to increase the uptake of HIV screening (Corbett *et al*., 2006; Setswe, 2009; International Labour Organization (ILO), 2015) and improve access to ART (Mahajan *et al*., 2007), our results show that stigma and discrimination still exist in some agricultural settings. Stigma and discrimination have been found to impede the progress of HIV responses (Chesney and Smith, 1999). Fear of stigma and discrimination can discourage workers from using HIV services and undermine interventions that seek to improve access to HIV services.

This study also found several positive experiences that improved access to HIV services. First, most workers could access HIV prevention services, mainly condoms, from their places of work. Secondly, the presence of peer educators and CHWs at the farms, and strengthening of support groups for people living with HIV, has been reported to be successful intervention in reducing access barriers (Garchitorena *et al*., 2021; Harwell *et al*., 2022). Evidence from the literature shows the effective role of support groups in addressing stigma-related factors and in reducing fear of stigma and discrimination among people living with HIV (Mburu *et al*., 2013; Lifson *et al*., no date). Lastly, cross-border migrant workers who are on ART could continue with treatment while travelling to their countries. Linkage to care is essential in HIV control as it ensures that optimal treatment outcomes are maintained (Bernardo *et al*., 2021), especially for cross-border farm workers.

Most studies fail to address workers’ health within the context of public health, but rather only focus on work-related diseases and injuries when addressing worker’s health. However, work in itself is a social determinant of health, and it contributes to disparities in health (Burgard and Lin, 2013; Benach *et al*., 2014). Farm workers are an interesting population because although they may encounter health access challenges similar to those of rural populations, they face unique employment conditions that contribute towards healthcare access challenges, which sets them apart from the general rural population. The unfavourable circumstances under which they are employed, combined with their educational level and economic background, make them a unique population group that requires unique approaches for their access to healthcare. Although HIV may not be an occupational disease for agricultural workers, all conditions associated with working in the sector expose these vulnerable workers to restricted access to HIV and other healthcare services.

Our main limitation is that this is a qualitative study, and thus the results may not be generalizable. However, efforts to ensure the credibility and trustworthiness of the findings were made through continuous researcher reflexivity throughout the study, coupled with data triangulation and the involvement of a second researcher in data analysis. The first strength of this study is the sampling method for IDIs, which allowed the sample to represent views of different demographic categories of farm workers. This sampling method ensures transferability to other settings with similar contexts.

The second and main strength of this study is the new concept reported on the SEM model, which had not been reported in previous studies. While previous studies have used the model to examine access to healthcare for certain population groups, they have never reported work as a determinant of accessing healthcare, particularly for vulnerable working groups. To the best of our knowledge, this is the first study to report on working conditions as a barrier to accessing HIV healthcare using the SEM model. As such, this study extends its contribution to the SEM model in studying access to healthcare for vulnerable working populations.

## Conclusion

This study provides a deeper understanding of the structural causes of poor HIV diagnosis and treatment coverage among farm workers. To our knowledge, this is the first study that connects work as a determinant of health to the structural causes of poor access to HIV health services. Farming is one of the key economic drivers in South Africa; therefore, inadequate access to HIV services for farm workers compromises HIV diagnosis and perpetuates the spread of HIV within the study population. This results in increased HIV-related morbidity and mortality among farm workers. Based on the findings of this study, we recommend that models of HIV care delivery for farm workers should be uniquely designed to address the identified access barriers, with more consideration of increasing mobile healthcare services and CHWs. We further recommend a review of agricultural sector working conditions, particularly those relating to health and HIV workplace policies. We also recommend that working hours and leave benefits be reviewed to ensure that all workers have equal access to healthcare. Overcoming health inequities and achieving UHC in HIV services cannot be successfully realized without the full participation of employers, particularly for vulnerable workers who are faced with several socio-economic determinants of health.

## Data Availability

The data underlying this article are available in the article. The interview data underlying this article cannot be shared publicly to maintain the privacy of the individuals who participated in the study.

## Reflexivity statement

This study involved two females and two male researchers with varying levels of seniority and experience in public health research. The lead author is a female doctoral student in the Global Health Department, at Stellenbosch University, while the co-authors hold senior and professorship positions in different academic institutions. All authors have extensive research experience within South Africa and other SSA countries.
